# Polyacrylamide injection matrix for serial femtosecond crystallography

**DOI:** 10.1038/s41598-019-39020-9

**Published:** 2019-02-21

**Authors:** Jaehyun Park, Sehan Park, Jangwoo Kim, Gisu Park, Yunje Cho, Ki Hyun Nam

**Affiliations:** 1 Pohang Accelerator Laboratory, Pohang, Gyeongbuk Republic of Korea; 2 Department of Life Science, POSTECH, Pohang, Gyeongbuk Republic of Korea; 3 Division of Biotechnology, Korea University, Seoul, Republic of Korea; 4 Institute of Life Science and Natural Resources, Korea University, Seoul, Republic of Korea

## Abstract

Serial femtosecond crystallography (SFX) provides opportunities to observe the dynamics of macromolecules without causing radiation damage at room temperature. Although SFX provides a biologically more reliable crystal structure than provided by the existing synchrotron sources, there are limitations due to the consumption of many crystal samples. A viscous medium as a carrier matrix reduces the flow rate of the crystal sample from the injector, thereby dramatically reducing sample consumption. However, the currently available media cannot be applied to specific crystal samples owing to reactions between the viscous medium and crystal sample. The discovery and characterisation of a new delivery medium for SFX can further expand its use. Herein, we report the preparation of a polyacrylamide (PAM) injection matrix to determine the crystal structure with an X-ray free-electron laser. We obtained 11,936 and 22,213 indexed images using 0.5 mg lysozyme and 1.0 mg thermolysin, respectively. We determined the crystal structures of lysozyme and thermolysin delivered in PAM at 1.7 Å and 1.8 Å resolutions. The maximum background scattering from PAM was lower than monoolein, a commonly used viscous medium. Our results show that PAM can be used as a sample delivery media in SFX studies.

## Introduction

X-ray free-electron laser (XFEL) is a newly emerged X-ray source with unprecedented levels of brilliance, excellent spatial coherence, and ultrashort pulse durations^1^. Serial femtosecond crystallography (SFX) using XFEL provides a new opportunity to visualise the crystal structures of macromolecules at room temperature without facilitating radiation-damage based on the ‘diffraction before destruction’ regime^2–5^. This technique not only provides biologically more reliable crystal structures but also molecular dynamics with a time-resolved study using ultrafast X-ray pulses^2–5^.

In the first stages of SFX experiments, individual crystal samples are delivered to the X-ray interaction point *via* a liquid jet injector using gas dynamic virtual nozzle (GDVN)^6^. This method successfully delivers the crystal sample to the X-ray pulse and reveals the crystal structure at high resolution^7^; however, the consumption of many crystal samples in SFX remains an experimental challenge. To reduce the sample consumption, other sample delivery methods, such as electrospinning^8^, LCP (lipidic cubic phase) injectors^9,10^, acoustic injectors^11^, or fixed-target scanning^12^, have been developed to deliver crystal samples continuously to the X-ray pulses in the SFX study. These methods reduce the flow rate of sample delivery from the injector or increase the hit-rate between the crystal sample and X-ray pulse^8–12^. Among the various methods to reduce the sample consumption, the method involving an injection matrix using a viscous medium drastically reduces the consumption of crystal samples by lowering the flow rate of the sample loading from the injector^10,13–17^. The protein crystal structure has been successfully determined using monoolein^9,10^, grease^13^, agarose^14^, or carbohydrate-based^15–17^ and polymer-based^17^ media as the injection matrix^10,13–17^. These delivery media are useful in SFX studies; however, there are cases in which the sample protein cannot be used due to chemical reactions or physical effects. For example, when lipid-binding proteins or cellulase are mixed with monoolein or a cellulose-based matrix, respectively, as the sample delivery material, the crystal suspension may weaken or dissolve due to reactions between the protein and delivery media. Therefore, the discovery and characterisation of new delivery matrix materials could provide a methodically extended opportunity to perform SFX studies.

Polyacrylamide (PAM) is a non-toxic polymer (-CH_2_CHCONH_2_-) formed from an acrylamide subunit, which is a highly water-soluble vinyl monomer^18^. Cross-linked PAMs are water-insoluble, and form a soft gel in the presence of water^19^. PAM exhibits stability over a wide range of pH interval (pH, 3–11) separating proteins, nucleic acids, and other biomolecules, and has been widely applied in selective media techniques in electrophoresis^20^. In particular, PAM is widely used in native gel electrophoresis because it has no non-specific or specific binding interactions with proteins^21^.

Herein, we report the preparation of a PAM injector matrix and characterisation of the stable injection conditions for SFX. We determined the crystal structures of lysozyme and thermolysin at 1.7 Å and 1.8 Å resolutions, respectively, using the PAM injection matrix at the Pohang Accelerator Laboratory X-ray Free Electron Laser (PAL-XFEL) facility.

## Results and Discussion

### Preparation of the polyacrylamide injection matrix for serial femtosecond crystallography

**Figure 1:**
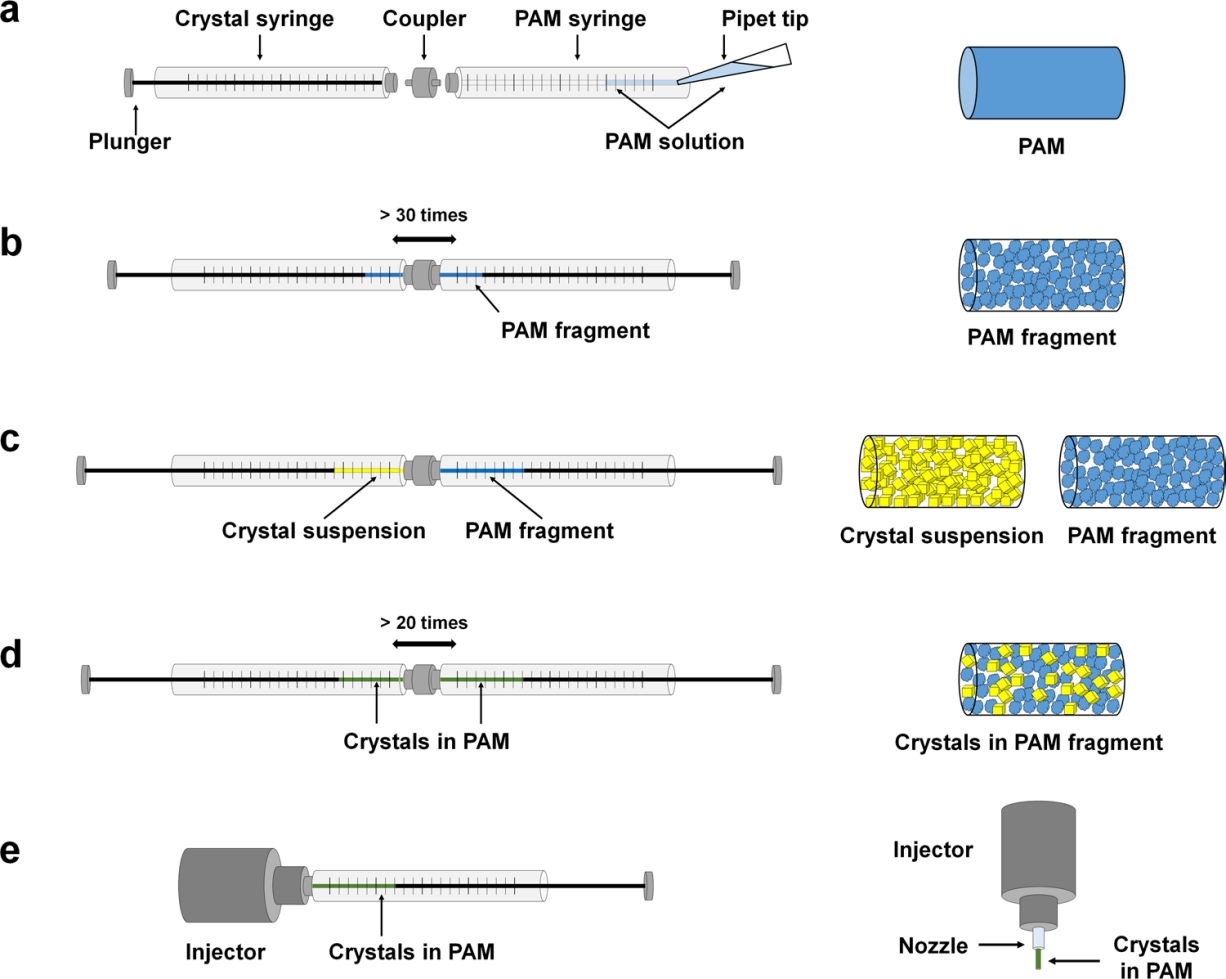
Preparation of the polyacrylamide injection matrix for serial femtosecond crystallography. (**a**) The polyacrylamide (PAM) solution is transferred to the syringe and further polymerised. (**b**) After the syringe-mixing setup, dispense repeatedly for more than 30 times to generate the PAM fragments. (**c**) Transfer the PAM fragments to one syringe and the crystal suspension to another syringe. (**d**) Dispense the two syringes gently >20 times to mix the PAM and crystal sample. (**e**) Transfer the mixture of crystals and PAM to the injector.

The syringe-setup method was used to mix the crystal sample with PAM, as previously reported with LCP and agarose media^9,14^. However, additional efforts were required to avoid damage to the crystal before mixing (Fig. 1). Polymerisation of PAM is initialised by adding ammonium persulphate and N,N,N′,N′-tetramethylethylenediamine (TEMED), which generates thermal heat during the polymerisation process^20^. These potential interstitials can have a negative physical impact on protein and crystal lattices. First, the solution was placed in a syringe and polymerisation was preferentially allowed for 10 min (Fig. 1a). For the SFX experiment, we used the final 10% (w/v) PAM injection matrix, which was prepared at a ratio of 1:1 of 20% (w/v) PAM and the protein crystal suspension. The 20% (w/v) PAM has high strength and could physically damage the crystals when mixed directly with a crystal sample, thereby resulting in reduced diffraction intensity with high mosaicity. To prevent physical damage to crystal, the PAM fragments were initially produced by dispense repeating over 30 times in the syringe setup (Fig. 1b). After the PAM fragment was transferred to one syringe, the coupler and other syringe containing the crystal suspension were connected (Fig. 1c). The PAM and crystal suspension were gently dispensed more than 20 times to evenly distribute the crystal samples in the PAM fragments (Fig. 1d). The mixed crystal sample embedded in the PAM fragments was transferred to the injector for the SFX experiment (Fig. 1e). Therefore, we used the viscosity of the PAM fragment to deliver the crystal sample by placing the crystals between the PAM fragments.

### Screening of the polyacrylamide matrix injection

The delivery medium for SFX experiment should keep the crystal sample stable and provide a continuous and stable stream when ejecting from the injector^14^. In order to find a condition in which the crystal sample provides a stable stream from the injector, we screened the delivery conditions according to the PAM concentrations.

**Figure 2:**
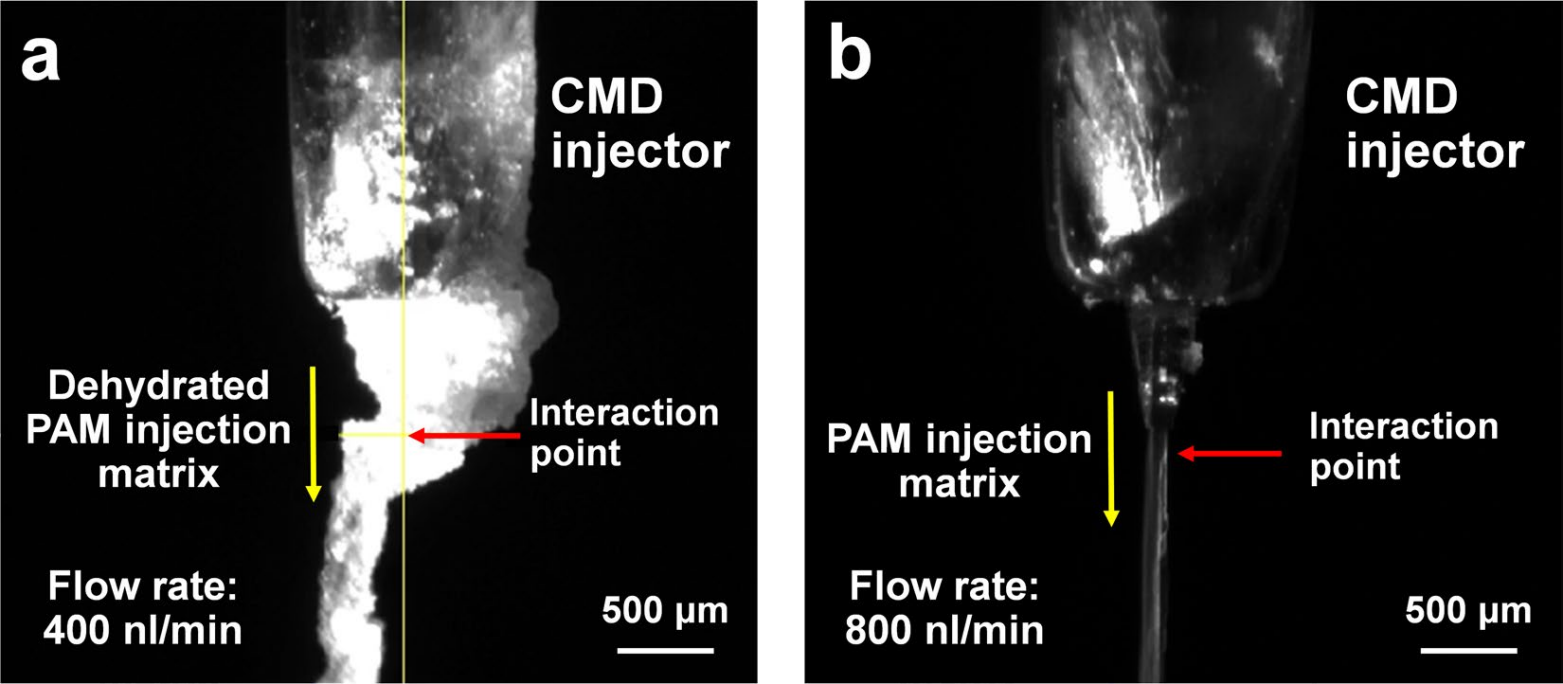
Injection of the polyacrylamide matrix from CMD injector. Snapshot of the stream of PAM injection at flow rate of (**a**) 400 nl/min and (**b**) 800 nl/min.

The PAM matrix was loaded at a flow rate of 500 nl/min through the 100 μm internal diameter (ID) nozzle of a CMD injector. The 5–8% (w/v) PAM from the injector generated droplets hanging from the end of the nozzle instead of a stable stream whereas 9–15% (w/v) PAM generated a stable stream (Supplementary Movie S1). Next, we screened the suitable flow rate for the SFX experiment for the final 10% (w/v) PAM injection matrix. The PAM injection matrix was guided into the X-ray interaction point by helium flow (focusing gas pressure: 1–2 × 10^5^ Pa) from the outer capillary of the injector. The PAM matrix was delivered at a stable flow rate of <400 nl/min (Fig. 2a). However, strong background scattering, originating from the PAM, was observed at approximately 4 Å (Supplementary Figure S1). This was caused by the helium gas flowing from the outer capillary of the injector as the PAM was dehydrated (Fig. 2a). Dehydration of PAM by helium gas focusing occurred both in air or helium ambience inside the sample chamber. We increased the flow rate of the PAM matrix from the injector to prevent the dehydration of the PAM matrix. The background scattering from the PAM could be removed at a flow rate of more than 800 nl/min (Fig. 2b). During SFX data collection, the crystal samples were delivered to the XFEL interaction point at a flow rate of 800 nl–2 μl/min (stream velocity: 1.69–4.24 mm/s).

### Serial femtosecond crystallography using the polyacrylamide injection matrix

To demonstrate the application of PAM injection matrix, we performed the SFX study using the lysozyme and thermolysin as model samples. The lysozyme crystal size was 30 × 30 × 30 μm^3^ and number density was approximately 6.7 × 10^7^ crystals/ml (Supplementary Figure S2). We collect a total of 25,603 images during the SFX experiment at room temperature. During the diffraction image collection, we used a total crystal volume of 10 μl with consumption of 0.5 mg lysozyme. The image containing 20 or more Bragg peaks was filtered out by the Cheetah program^22^ with the peakfinder8 algorithm and obtained 17,675 hit images with a hit rate of 69%. The diffraction images were indexed by CrystFEL^23^, which converged the input cell parameters with tolerance as the default value. The initial processed data were re-indexed using the optimised detector geometry. A total of 11,936 indexed images were obtained with an indexing rate of 67.52% (Supplementary Figure S3). The number of unique reflections was 14,007. Post-refinement was performed using the *partialator* with model unity. The overall signal-to-noise ratio (SNR) was 4.32 with 100% completeness. The overall R_split_ and Pearson correlation efficiency (CC*) were 15.31% and 99.2%, respectively (Table 1).

**Table 1:** Data collection and refinement statistics.

Data collection	Lysozyme	Thermolysin
Energy (eV)	9700	9692
Photons/pulse	~2 × 10^11^	~2 × 10^11^
Pulse width^a^	20 fs	20 fs
Space group	P4_3_2_1_2	P6_1_22
Cell dimensions		
*a*, *b*, *c* (Å)	78.61, 90.55, 73.42	92.66, 92.66, 128.59
No. collected diffraction images	25603	221595
No. of hits	17676	47865
No. of indexed images	11936	22213
No. of unique reflections	14007 (1353)	30878 (3028)
Resolution (Å)	80.0–1.70 (1.76–1.70)	131.57–1.80 (1.86–1.80)
Completeness	100.0 (100.0)	100.0 (100.0)
Redundancy	965.3 (676.7)	663.8 (471.0)
*I/σ(I)*	4.32 (1.39)	4.67 (1.16)
*R*_split_^b^	15.31 (73.37)	14.63 (84.09)
CC	0.970 (0.644)	0.975 (0.617)
CC*	0.992 (0.885)	0.993 (0.874)
Wilson B factor (Å^2^)	38.88	33.18
**Refinement statistics**		
Resolution (Å)	26.04–1.70	38.3–1.80
R_factor_/R_free_ (%)^c^	19.61/21.74	17.27/20.24
B-factor (Averaged)		
Protein	45.32	37.66
Metal	45.80	29.57
Water	46.20	41.00
Product		40.22
R.m.s. deviations		
Bond lengths (Å)	0.008	0.011
Bond angles (°)	0.94	1.01
Ramachandran plot (%)		
favored	97.64	96.82
allowed	2.36	3.18

Highest resolution shell is shown in parentheses.

^a^Electron bunch length.

^b^$${R}_{split}=(1/\sqrt{2})\cdot \frac{\sum _{hkl}|{I}_{hkl}^{even}-{I}_{hkl}^{odd}|}{\tfrac{1}{2}|{I}_{hkl}^{even}-{I}_{hkl}^{odd}|}$$.

^c^*R*_work_ = Σ||*F*_obs_| − |*F*_calc_||/Σ|*F*_obs_|, where *F*_obs_ and *F*_calc_ are the observed and calculated structure-factor amplitudes respectively. R_free_ was calculated as R_work_ using a randomly selected subset (10%) of unique reflections not used for structure refinement.

**Figure 3:**
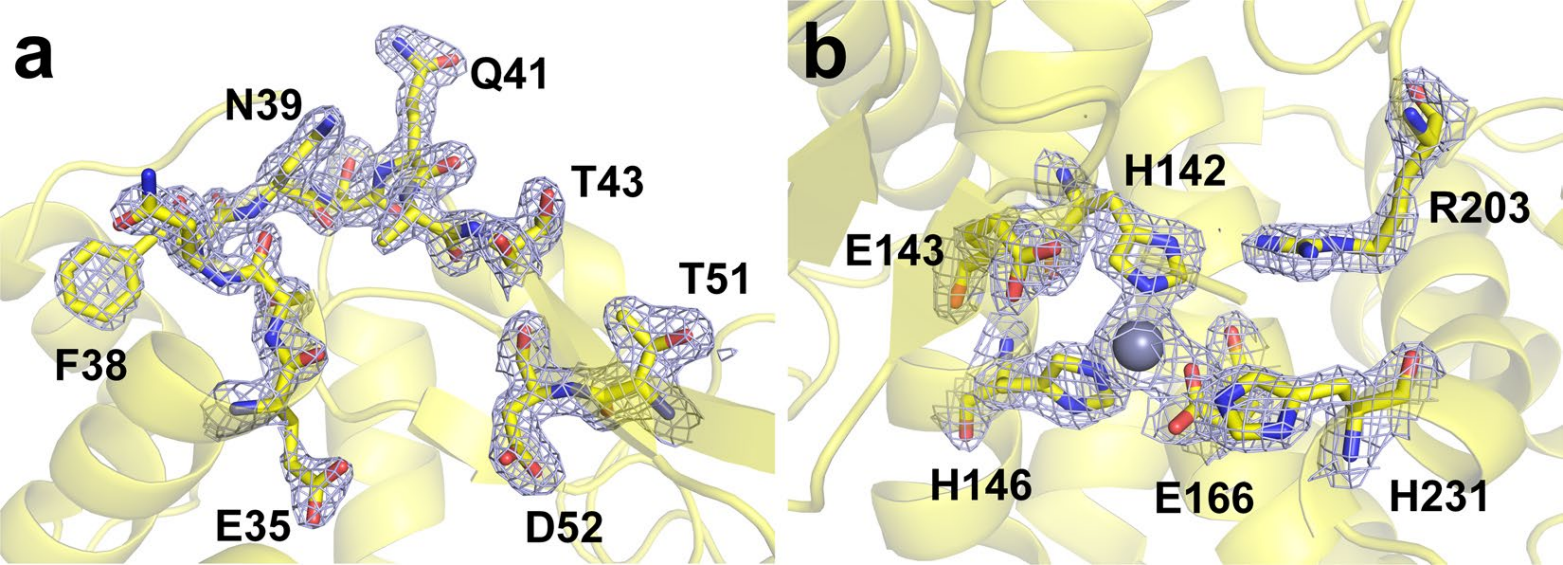
Electron density maps of lysozyme and thermolysin delivered in the polyacrylamide injection matrix. The 2Fo-Fc electron density maps of (**a**) lysozyme (light blue mesh, 1.2 σ) and (**b**) thermolysin (light blue mesh, 1.5 σ).

The resolution of the lysozyme structure was 1.7 Å, and the R_work_ and R_free_ were 19.61% and 21.74%, respectively. The electron density map was very clear for the interpretation of amino acids from Lys1 to Leu129. The catalytic amino acids (Glu35 and Asp52) in the active site of the lysozyme were well-refined (Fig. 3a). In addition, the disulphide bond in the crystal structure was observed without radiation damage (Supplementary Figure S4). The superimposition of the lysozyme delivered in PAM structure with previously reported lysozyme structure at room temperature using the liquid jet injector using GDVN (PDB code 4ET8)^7^ and droplet injector (5DM9)^24^ revealed a high similarity with a root-mean-square (rms) deviation of <0.1691 Å for all Cα atoms (Supplementary Figure S5).

To confirm the applicability of PAM to various samples, we also determined the structure of thermolysin using PAM. The data processing progress of thermolysin was identical with that of lysozyme. The thermolysin crystal size was approximately 8 × 8 × 200 μm^3^ and the number density was approximately 3.6 × 10^7^ crystals/ml (Supplementary Figure S2). We broke the rod-shape thermolysin crystal into small crystals using a pipet tip in a microcentrifuge tube to avoid clogging of the injector nozzle. The size of thermolysin crystals used in the experiment was approximately 8 × 8 × 8 μm^3^ (Supplementary Figure S2). Total 228,304 images of thermolysin images were collected at room temperature. During the diffraction image collection, we used a 40 μl crystal volume with consumption of 1.0 mg thermolysin. A total of 47,865 hit images were obtained with a hit rate of 20.96%. After the optimisation of detector geometry, 22,213 indexed images were obtained with an indexing rate of 50.9% (Supplementary Figure S6). The number of unique reflections was 30878. The overall SNR was 4.67 with 100% completeness. The overall R_split_ and CC* were 14.63% and 99.3%, respectively (Table 1). The resolution of the thermolysin structure was 1.8 Å, and the R_work_ and R_free_ were 17.27% and 20.24%, respectively. The electron density map was very clear for the interpretation from Ile1 to Lys316. The zinc ion on the active site of the thermolysin was coordinated by His142, His146, and Glu166 (Fig. 3b). The calcium ions directly involved in the activity were observed without radiation damages (Supplementary Figure S7). The superimposition of the thermolysin delivered in the PAM structure with the previously reported thermolysin structure at room temperature using the nanoflow liquid injector (PDB code 4OW3)^24^, acoustic injectors for drop-on-demand (5HQD)^11^, synthetic grease super lube (5WR2)^25^, and hydroxyethyl cellulose (5WR3)^25^ revealed a high similarity with a rms deviation of 0.1902–0.3439 Å for all Cα atoms (Supplementary Figure S8).

Although the overall structure of thermolysin derived in PAM is similar to that of the previously reported thermolysin structure, there were two distinct differences in the electron density map. First, a dipeptide (Leu-Lys) was found at the substrate recognition site of thermolysin (Supplementary Figure S9). Although this dipeptide is not found in other SFX thermolysin structures, it is often found in the crystal structures determined using a synchrotron^26^. This dipeptide presumably corresponds to the substrate-cleaved product^26^. Second, we observed a strong Fo-Fc electron density map (>5 σ) around the zinc binding site in the active site of thermolysin (Supplementary Figure S9). The identity of the substance in this density map is unclear; however, we are currently conducting an analysis to identify the substance since it can assist in the study of the function of thermolysin on a latent basis.

### Measurement of background scattering

**Figure 4:**
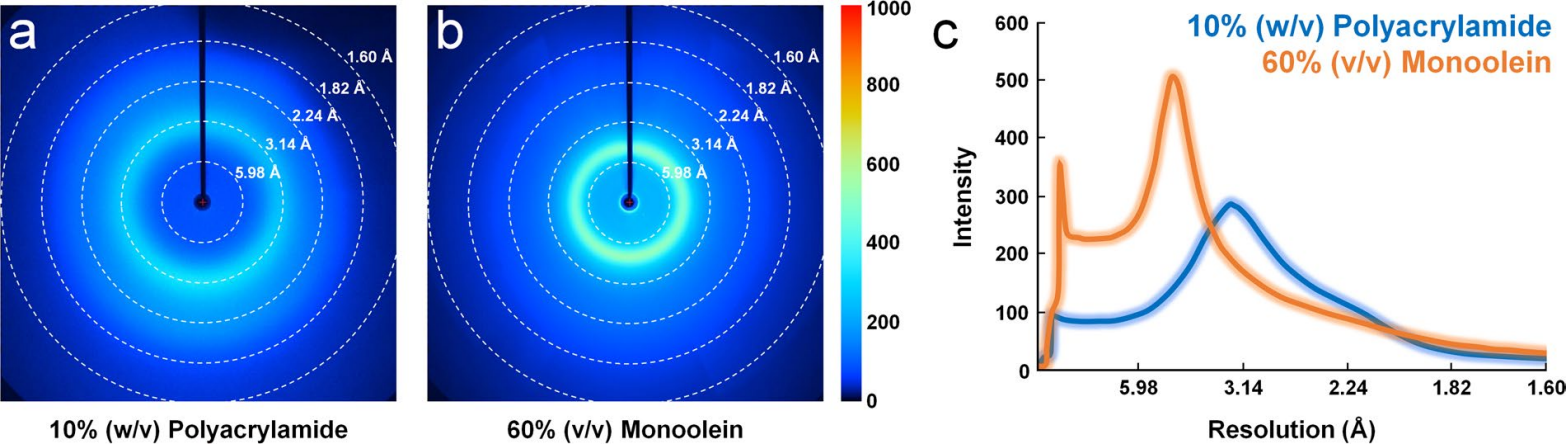
Comparison between the background X-ray scattering of polyacrylamide and monoolein. The average background scattering intensities of approximately 1,000 images from (**a**) 10% (w/v) polyacrylamide and (**b**) 60% (v/v) monoolein (**c**) The two-dimensional profile of the scattering intensities of polyacrylamide (blue) and monoolein (red).

The X-ray scattering signal is generated from the delivery medium as well as the diffraction signal of the crystal sample. Minimising the X-ray scattering generated from the delivery medium plays an important role in improving the SNR of the diffraction pattern during data processing. The delivery mediums for SFX used up to now provide a unique X-ray scattering signal for a specific angle^16^. Analysis of this background scattering provides an important information for selecting the delivery material considering the diffraction limit of the crystal sample. We compared the X-ray scattering measurements of PAM with monoolein, which is widely used in SFX research. 10% (w/v) PAM and 60% (v/v) monoolein were injected at a flow rate of approximately 1 μl/min from a 100 μm ID nozzle. We extracted the hit images only in injection stream during the SFX data collection using an X-ray pulse with approximately 2 × 10^11^ photons/pulse. The average scattering intensity was obtained from 1,000 images considering the intensity fluctuation of XFEL, and the scattering signal from PAM and monoolein were analysed from the beam centre to 1.6 Å (Fig. 4).

PAM exhibited diffuse scattering at a resolution of 3.2 Å, which was considered to have originated from a solvent containing water and not the PAM matrix (Fig. 4a). The average maximum scattering intensity from the solvent was approximately 275 ADU. Monoolein, on the other hand, exhibited diffuse scattering at 4.5 Å resolution, which was considered to be originated from the lipid chain (Fig. 4b). The average maximum scattering intensity from the monoolein was approximately 510 ADU. Therefore, PAM in the maximum scattering region exhibited less background scattering than monoolein (Fig. 4c). On the other hand, since the flow rate of the PAM injection matrix was higher than that of monoolein, the sample consumption was relatively high in the former when the same data was collected. In this experiment, we tried to increase the flow rate to prevent the dehydration of the PAM injection matrix. The addition of a viscous substance such as glycerol, which does not react with protein crystals could render the PAM injection matrix to be used for SFX at a low flow rate by delaying the dehydration. Further, the use of nozzles with a narrow ID suitable for a crystal sample could reduce the sample consumption.

Next, we compared the PAM with previously reported hydrophilic injection matrices. We found very weak diffused background scattering at 2.6–4 Å, which centred on the scattering peak that is considered to be water at 3.2 Å. These features are very similar to hydroxyethyl cellulose, hyaluronic acid, agarose, and carboxymethyl cellulose, and commonly exhibit low diffuse scattering. These delivery materials are commonly considered to be in an irregular arrangement in the gel state, resulting in weak background scattering. On the other hand, hydrogel Pluronic F-127 showed relatively wide and strong scattering intensity at 2.8–5 Å.

### Chemical compatibility

In previous hydrophilic injection matrix studies, the hydrogel showed a change in viscosity with high concentration crystallization precipitation^14,17^. These precipitants can potentially affect the stability of the injection stream^14,17^. To better understand the molecular properties of the PAM by crystallization precipitation, we performed chemical compatibility studies between PAM and crystallization precipitants. When 10% (w/v) PAM mixed with 2.1 M NaCl and 2 M (NH_4_)_2_SO_4_, injection media provided a stable stream without the influence of viscosity. In contrast, when 10% (w/v) PAM were mixed with 25% (v/v) PEG 400, PEG 1000, PEG 4000, PEG 8000, and MPD, the injection media had low viscosity and did not provide stable injection stream. To increase the viscosity of the PAM injection matrix, we increased the concentration of PAM to 15%, and performed compatibility screening for PEG and organic liquids. The PAM injection matrix was stable at a final concentration of 10% (w/v) PEG 400, 7% (w/v) PEG 1000, 5% (w/v) PEG 4000, 5% (w/v) PEG 8000, or 10% (v/v) MPD. The viscosity of PAM can be increased by increasing the concentration of PAM; however, a final concentration of >20% (w/v) PAM is physically difficult to mix with the crystal sample in the syringe due to its high strength. Among various hydrophilic injection matrices, agarose^14^, carboxymethyl cellulose sodium salt (NaCMC)^17^, and hydrogel Pluronic F-127^27^ have been studied for chemical comparability with crystallization precipitants. When compared with previously reported hydrogels, PAM was very stably injected at higher (NH_4_)_2_SO_4_ concentrations of 2.0 M than agarose (1.25 M), NaCMC (1.8 M), and hydrogel Pluronic F-127 (0.25 M). In addition, PAM was stably injected at higher NaCl concentrations of 2.1 M than agarose (1 M), NaCMC (2 M), and Pluronic F-127 (2 M). In contrast, PAM was suitable for use as an injection matrix only when PEG 400–8000 and MPD were < 5–10%, whereas agarose (final > 30% PEG 400–8000)^14^, NaCMC [final 35% (w/v) PEG 400, 30% (w/v) PEG 2000, 25% (w/v) PEG 4000, 25% (w/v) Polypropylene glycol 400, and 25% (v/v) MPD]^17^, Pluronic F-127 [final 23% (w/v) PEG 400, 7% (w/v) PEG 2000, and 25% (w/v) Polypropylene glycol 400]^28^ was injected stably even with high concentration polymeric precipitants. Therefore, PAM can be used to suit crystallization conditions, including high salt or low polymer concentrations. Further studies to increase the viscosity of the PAM injection matrix are required for the use of polymer precipitants in high concentrations.

## Methods

### Crystallisation

The lysozyme from chicken egg white was purchased from Hampton Research (Cat No. HR7–110). The protein powder was dissolved into a buffer containing 10 mM Tris-HCl, pH 8.0 and 200 mM NaCl. The lysozyme solution (200 μl; 50 mg/ml) was mixed with the crystallisation solution (200 μl) containing 0.1 M sodium acetate, pH 4.0, 6% (w/v) polyethylene glycol 8000 and 10% (w/v) mM NaCl in 1.5 ml microcentrifuge tube, and subsequently mixed using a vortex machine at 3,000 rpm for 10 s. After incubation at 20 °C for 4 h, the supernatant of the mixture solution was removed to avoid the growing of the crystal size. Further, the crystal samples were stored in a solution containing 0.1 M sodium acetate, pH 4.0 and 0.2 M NaCl.

The thermolysin from *Bacillus thermoproteolyticus* was purchased from Hampton Research (Cat No. HR7-098). The protein powder was stored in deionised water for 20 minutes. NaOH (0.1 M) was added and the final thermolysin (25 mg/ml) solution was made into 50 mM NaOH. The protein solution was exchanged with a solution containing 10 mM Tris-HCl, pH 8.0 and 200 mM NaCl using a Centricon-30 YM filter (Millipore). The protein solution (100 mg/ml) was incubated at 4 °C, and microcrystals appeared after two months.

### Polyacrylamide preparation

Acrylamide/bis-acrylamide [500 μl; 20% (v/v)] solution, ammonium persulphate (5 μl; 15%), and TEMED (0.5 μl) were mixed in a microcentrifuge tube, and the PAM solution (50 μl) was transferred into a Hamilton syringe (81065-1710RNR). After the PAM solution was polymerised in the syringe, an empty syringe was connected using a coupler. To make the PAM fragments, the syringe plunger was moved back and forth more than 30 times, causing the PAM to pass through the narrow inner diameter of the coupler and break it. The PAM fragment was then transferred to one syringe, and the crystal suspension (50 μl) was loaded in the opposite syringe. PAM fragments and crystal suspensions were mixed with the plunger gently moving back and forth more than 20 times in the two coupled syringe set-up. The final percentage of the crystal pellet was approximately 20–25% in the total injection matrix. The crystal sample embedded in PAM fragments was transferred to the CMD (carrier matrix delivery) injector^29^ for the SFX experiment.

### Data collection

The diffraction data of the experiment using the PAM injection matrix with the X-ray pluses were collected at the NCI (Nano Crystallography and Coherence Imaging) experimental hutch at PAL-XFEL^28,30^. The X-ray energy was 9.7 keV (1.2782 Å) with a pulse energy of approximately 500 μJ and a repetition rate of 30 Hz. The photon flex was 1–2 × 10^11^ photons per pulse within 20 fs duration. The X-ray pulse was focused to 5 × 5 μm^2^ (FWHM) by a Kirkpatrick-Baez mirror^27^. The sample was delivered to the X-ray pulse on air (lysozyme) or helium (thermolysin) ambience at room temperature in the MICOSS (multifarious injection chamber for molecular structure study) system^29^. The diffraction data were collected on the MX225-HS (Rayonix, LLC) detector with a 4 × 4 binning mode (pixel size: 156 μm × 156 μm).

### Data processing and structure determination

The diffraction pattern was monitored online by OnDA^31^. The hit images were filtered by the Cheetah program^22^. The diffraction images were indexed, scaled, and merged by the CrystFEL program^23^. The phase problem of the lysozyme was solved by molecule replacement using the Phaser-MR in PHENIX^32^ with lysozyme (PDB code 4ET8)^7^ as the search model. The phasing of thermolysin was obtained by molecule replacement using the Phaser-MR in PHENIX^32^ with thermolysin (PDB code 5DM9)^24^ as the search model. The model building and refinement was carried out using the Coot^33^ and Phenix.refinement in PHENIX^32^. The geometric analysis was performed using the MolProbity^34^. The figures were generated by PyMOL (https://pymol.org/).

### Chemical compatibility tests

The chemical compatibility studies between PAM and the crystallization precipitants [(NH4)_2_SO_4_, NaCl, PEG 400, PEG 1000, PEG 4000, 2-methyl-2,4-pentanediol (MPD)] were performed at room temperature. A final 10% (w/v) or 15% (w/v) PAM was created using a solution containing 0.2 M Tris-HCl (pH 7.5). PAM (50 μl) and each precipitant (50 μl) were mixed using a two coupled Hamilton syringe. The viscosity of the mixture, and injection stream properties were judged visually based extruded behaviour. The mixture was extruded manually from a Hamilton syringe through a 168 μm inner diameter (ID) needle.

### Accession codes

The coordinates and structure factors have been deposited in the Protein Data Bank under the accession code 6IG6 (lysozyme) and 6IG7 (Thermolysin). Diffraction images have been deposited to CXIDB under ID 84 (Lysozyme) and 85 (Thermolysin).

## Supplementary information

Supplementary Movie S1

Supplementary data
