# Supplementary material for: Polyacrylamide injection matrix for serial femtosecond crystallography
Source: Sci Rep. 2019 Feb 21;9:2525. doi: 10.1038/s41598-019-39020-9 (PMC6385504; doi:10.1038/s41598-019-39020-9)
Supplement: Supplementary file 2 — Supplementary data [file 41598_2019_39020_MOESM2_ESM.docx]

**Supplementary Materials for**

**Polyacrylamide injection matrix for serial femtosecond crystallography**

Jaehyun Park^1,*^, Sehan Park^1^, Jangwoo Kim^1^, Gisu Park^1^, Yunje Cho^2^, Ki Hyun Nam^3,4,*^

^1^ Pohang Accelerator Laboratory, Pohang, Gyeongbuk, Republic of Korea.

^2^ Department of Life Science, POSTECH, Pohang, Gyeongbuk, Republic of Korea.

^3^ Division of Biotechnology, Korea University, Seoul, Republic of Korea.

^4^ Institute of Life Science and Natural Resources, Korea University, Seoul, Republic of Korea.

*Corresponding author.

Pohang Accelerator Laboratory, Pohang, Gyeongbuk, Republic of Korea.; E-mail: jaehyun.park@postech.ac.kr (J.P.)

Division of Biotechnology, Korea University, Seoul, Republic of Korea. E-mail: structures@korea.ac.kr (K.H.N.)


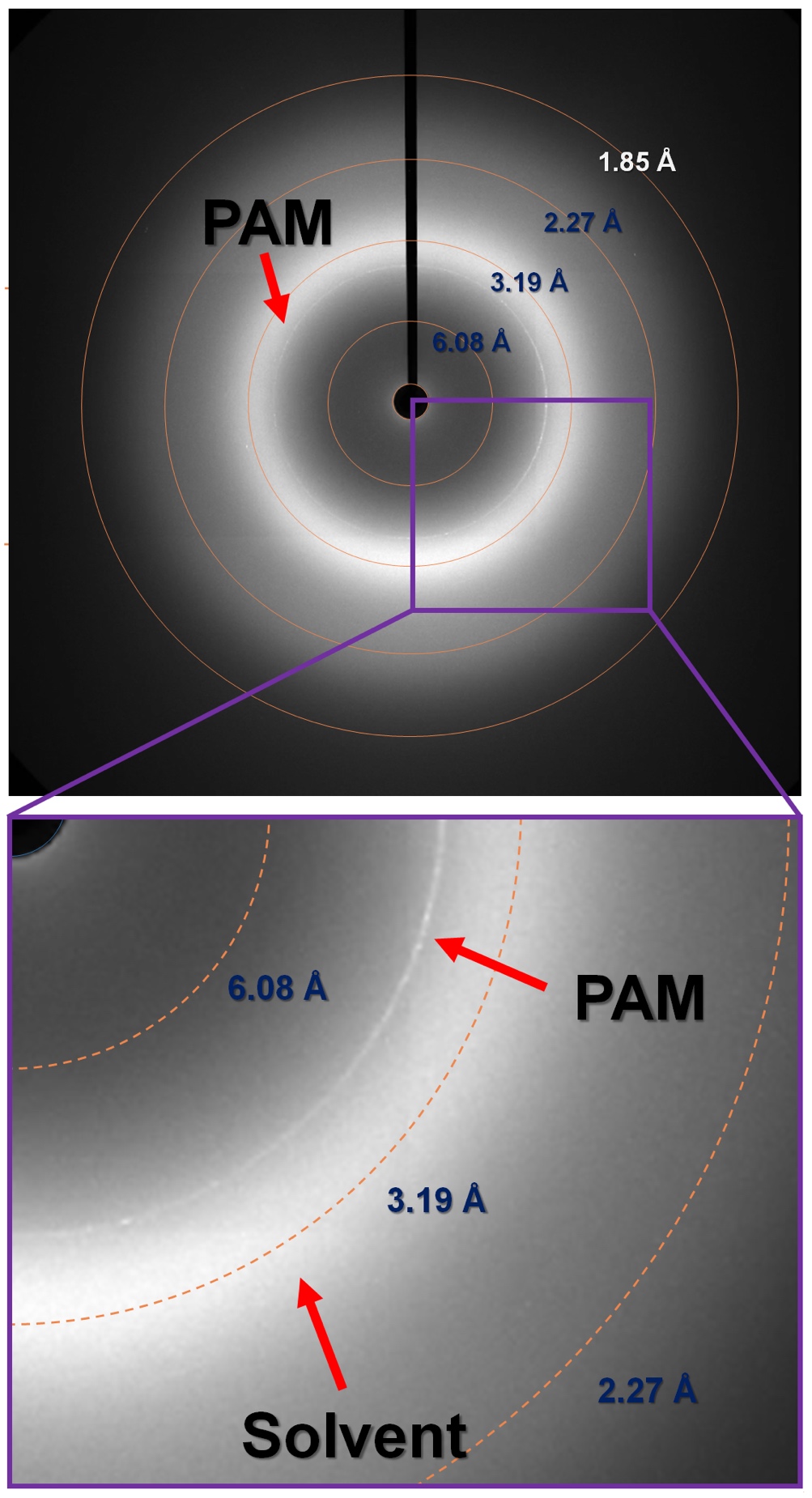


**Supplementary Figure S1.** Background scattering of the dehydrated 10% (w/v) polyacrylamide (PAM) injection matrix at flow rate of 400 nl/min. The dehydrated PAM shows the background scattering around 4 Å. The diffuse scattering from the solvent is observed at 3.3 Å.

**
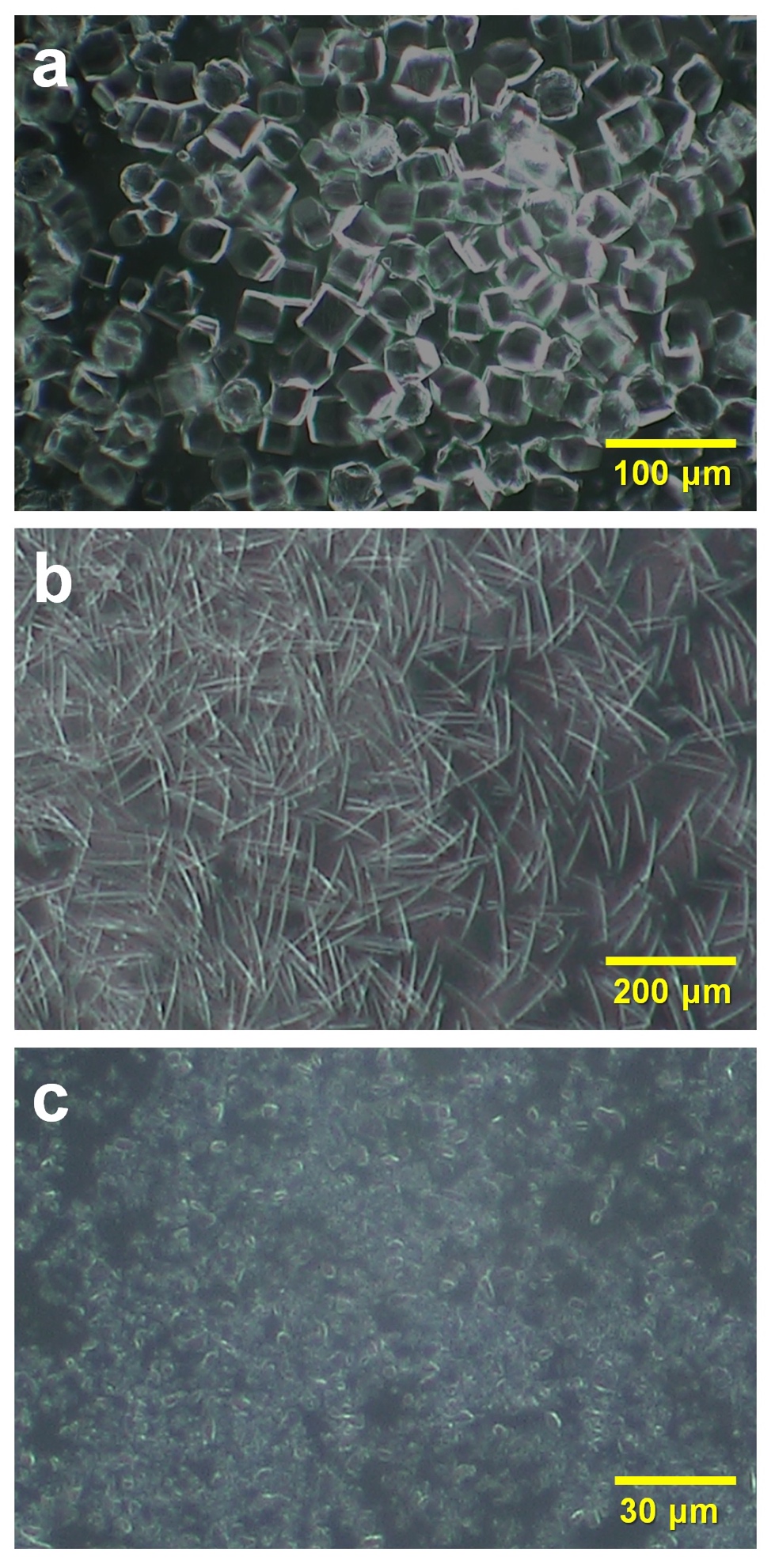
**

**Supplementary Figure S2.** Crystal photos of (**a**) lysozyme, (**b**) thermolysin, and (**c**) broken thermolysin.

**
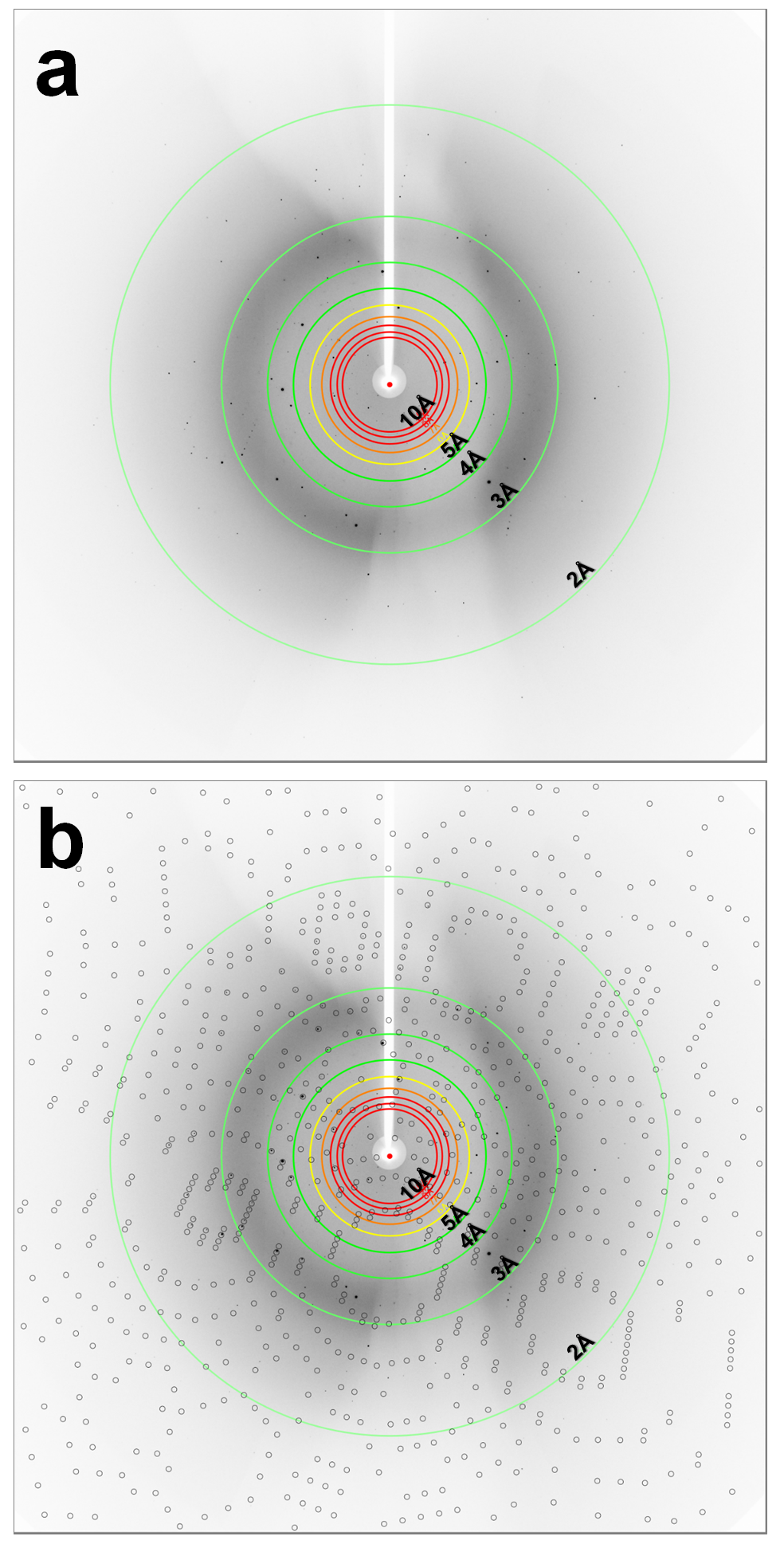
**

**Supplementary Figure S3.** (**a**) Diffraction pattern of lysozyme derived in polyacrylamide. (**b**) Indexed imaged of lysozyme derived in polyacrylamide.


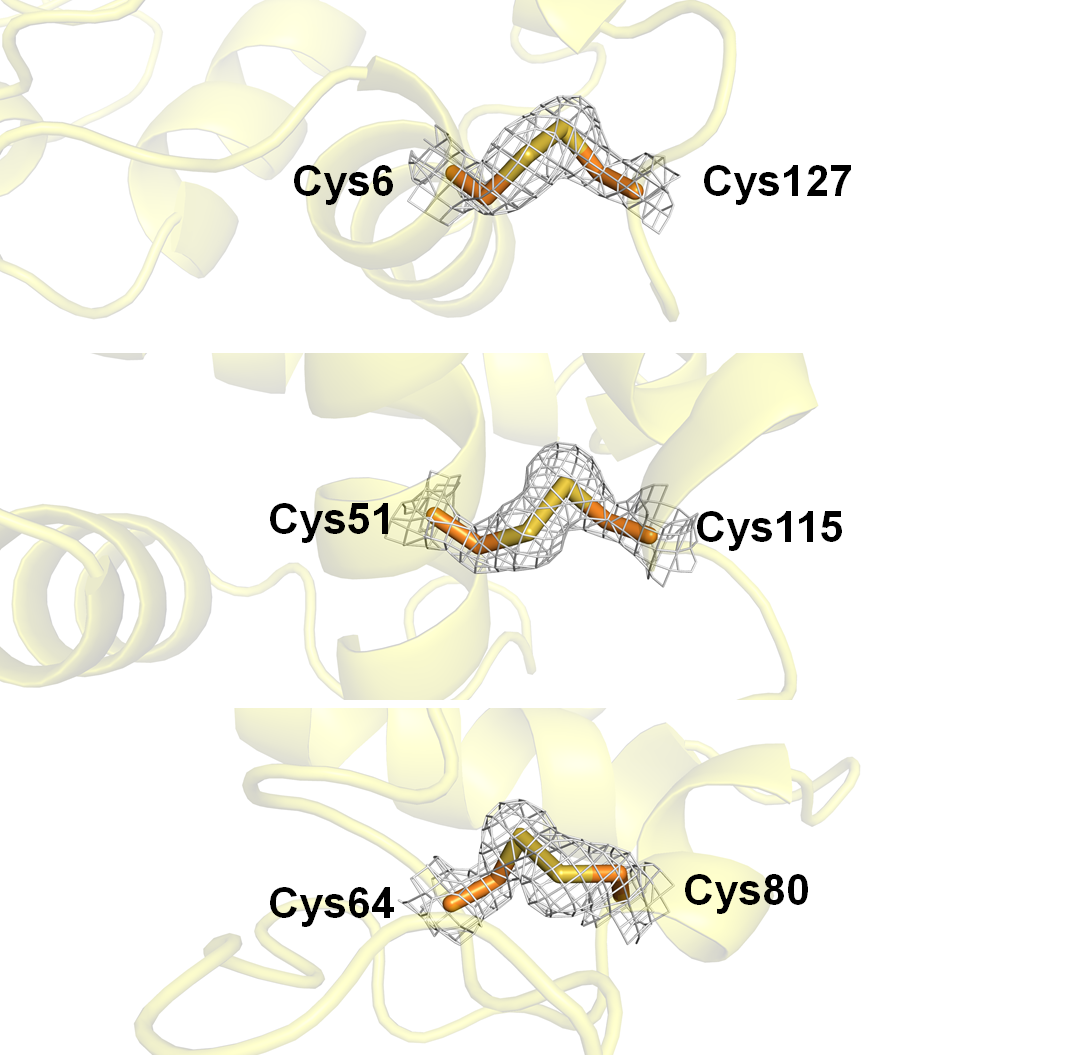


**Supplementary Figure S4**. The 2Fo-Fc electron density map (grey, counted 1.5 σ) of disulfide bond in the lysozyme delivered in polyacrylamide.


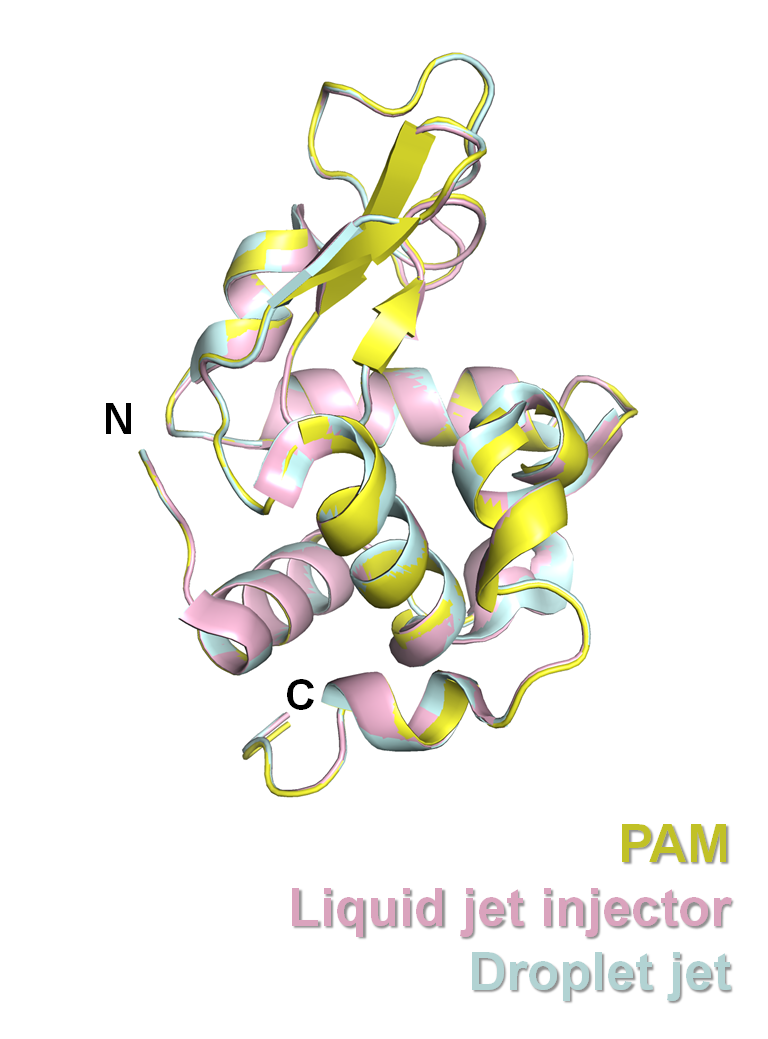


**Supplementary Figure S5.** Superimposition of the crystal structure of lysozyme delivered in polyacrylamide (PAM) with lysozyme delivered as liquid jet with Gas Dynamic Virtual Nozzle (PDB code: 4ET8, root-mean-square deviation of 0.1691) and droplet injector (5DM9, 0.1590).

**
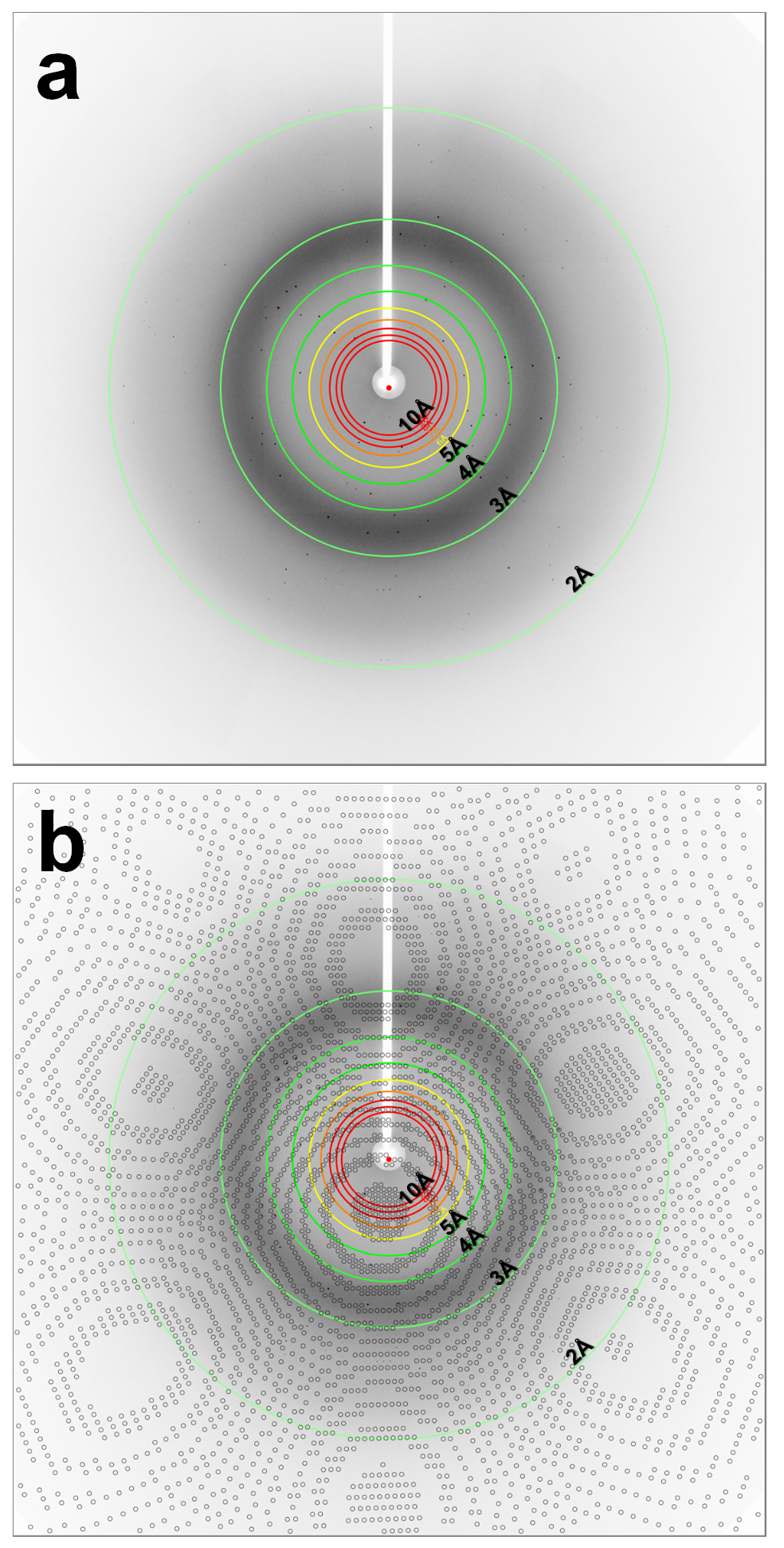
**

**Supplementary Figure S6.** (**a**) Diffraction pattern of the thermolysin derived in polyacrylamide. (**b**) Indexed imaged of the thermolysin derived in polyacrylamide.


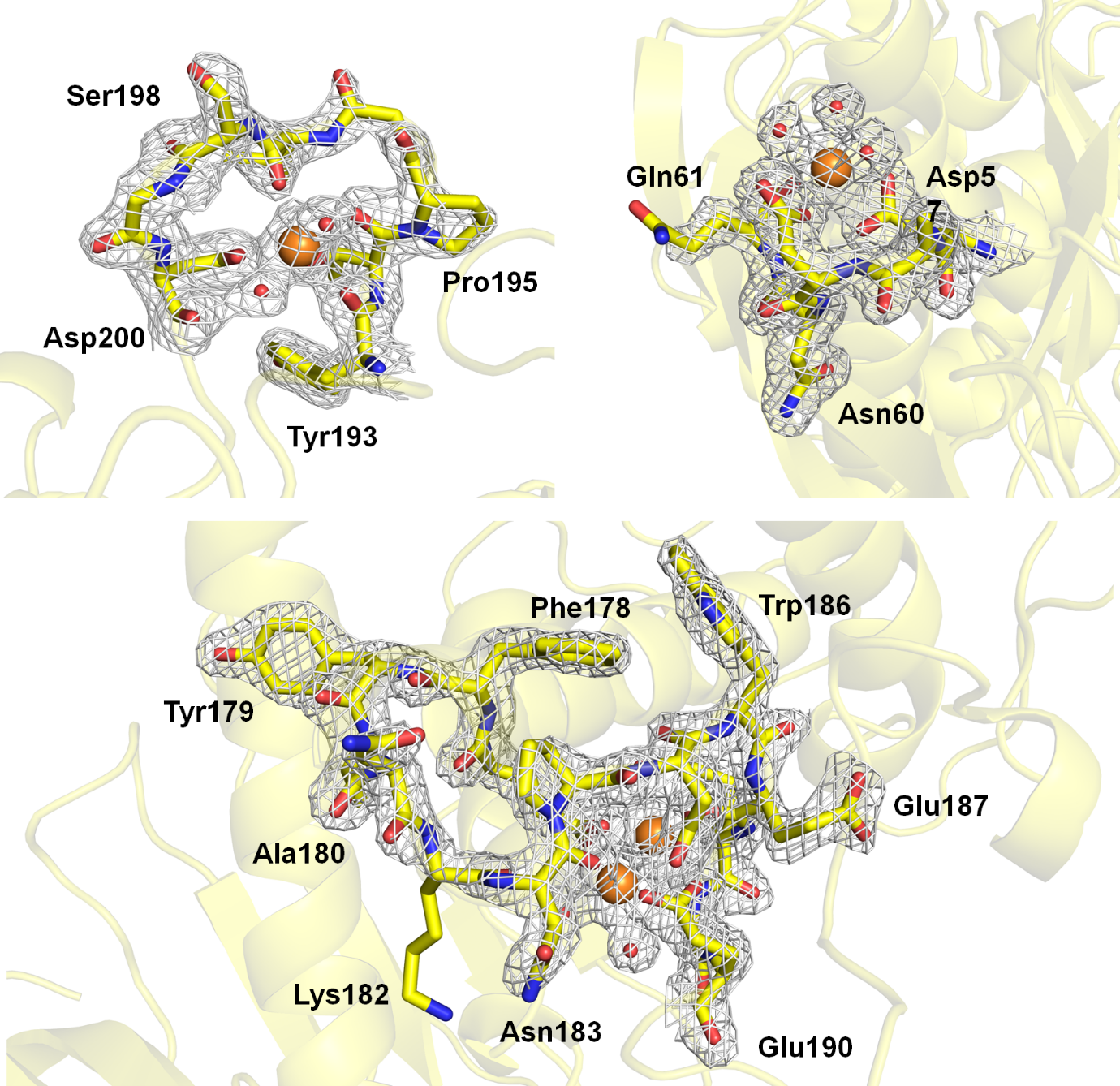


**Supplementary Figure S7**. The 2Fo-Fc electron density map (grey, counted 1.5 σ) of the Ca binding site of thermolysin derived in polyacrylamide.


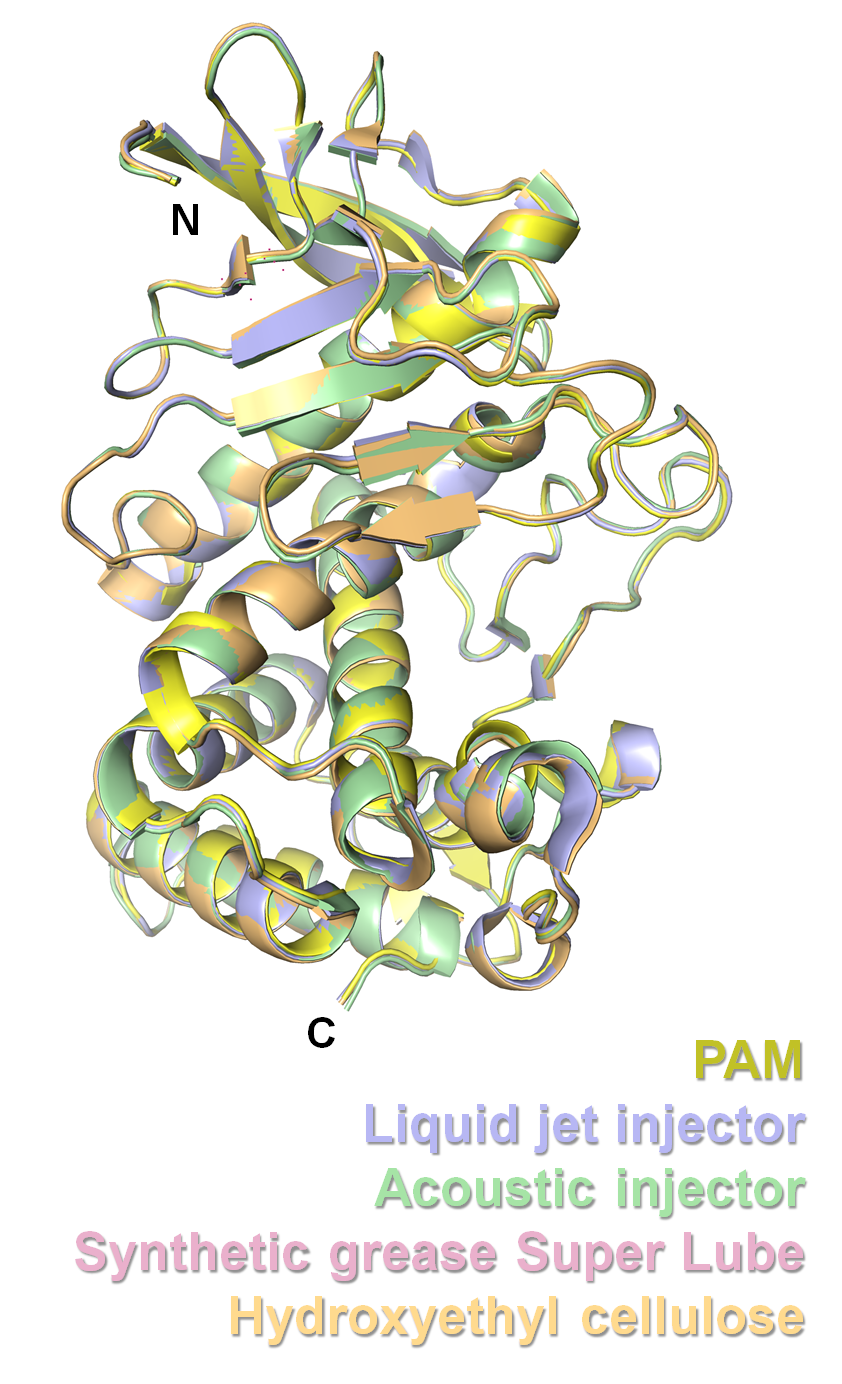


**Supplementary Figure S8.** Superimposition of the crystal structure of thermolysin delivered in polyacrylamide (PAM) with lysozyme delivered from the liquid jet as Gas Dynamic Virtual Nozzle (PDB code: 4OW3, root-mean-square deviation of 0.1902), acoustic injectors (5HQD, 0.3118), synthetic grease super lube (5WR2, 0.3439) and hydroxyethyl cellulose (5WR3, 0.3380).

**
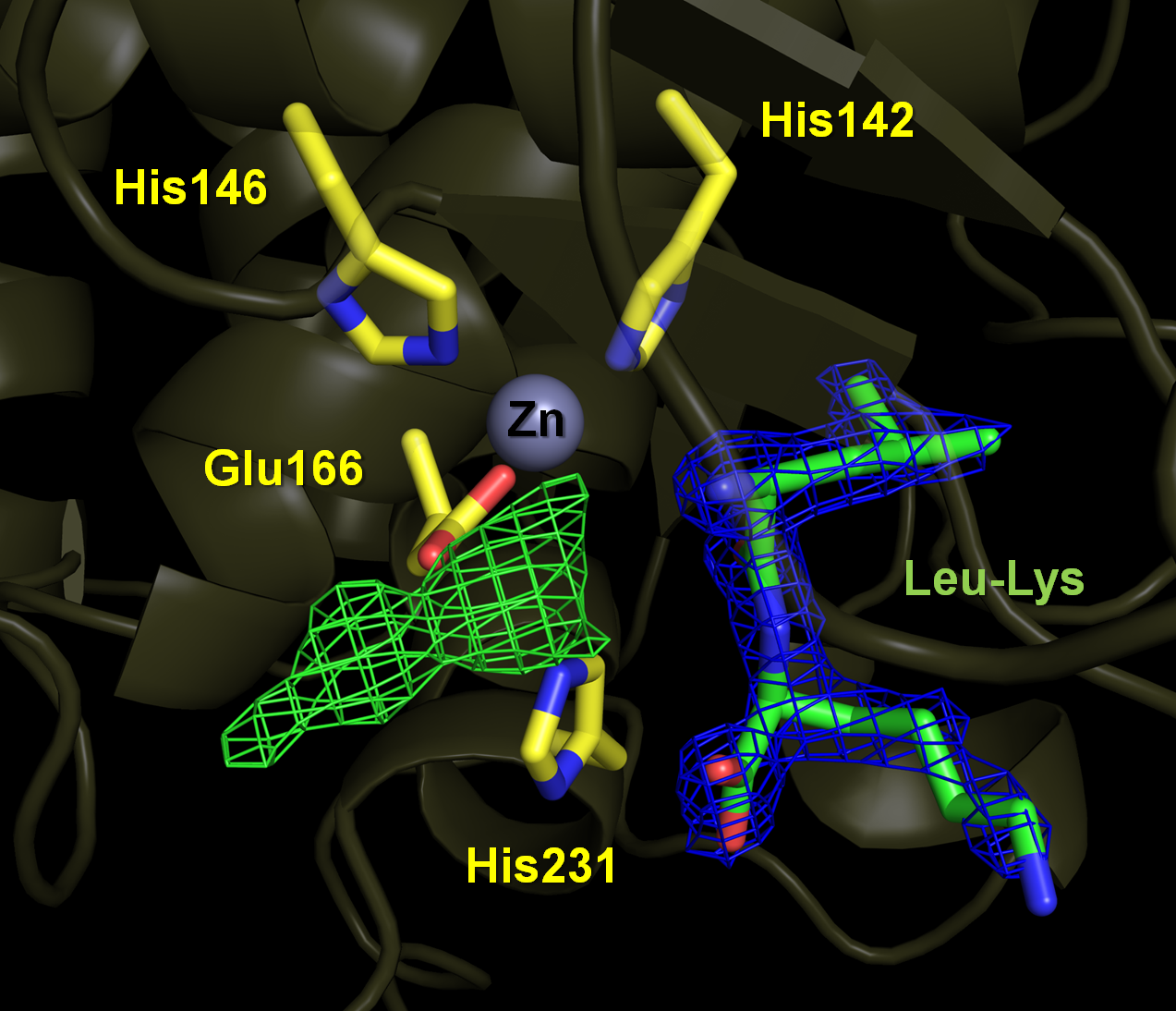
**

**Supplementary Figure S9.** Close-up view of the electron density map of thermolysin derived in polyacrylamide. A 2Fo-Fc electron density map (blue, counted at 1.5σ) for the dipeptide product Leu-Lys and an undefined Fo-Fc (green, counted at 5σ) electron density map are observed around the catalytic Zn binding site of thermolysin.
